# A versatile method for dorsal-approach plantar plate repair using standard operative instruments

**DOI:** 10.1186/s12891-021-04951-w

**Published:** 2022-01-03

**Authors:** Shun-Min Chang, Peng-Ju Huang, Chui Jia Farn, Shin-Yiing Lin, Chen-Chie Wang, Chung-Li Wang, Pei-Yu Chen

**Affiliations:** 1 Department of Orthopedics, Kaohsiung Municipal Ta-Tung Hospital, Kaohsiung Medical University, No.68, Jhonghua 3rd Rd, Cianjin District, Kaohsiung, 80145 Taiwan; 2 Department of Orthopedics, Kaohsiung Medical University Chung-Ho Memorial Hospital, No.100, Tzyou 1st Road, Kaohsiung, 807 Taiwan; 3 Department of Orthopedic Surgery, National Taiwan University Hospital, No.7, Chung Shan S. Rd. (Zhongshan S. Rd.), Zhongzheng Dist, Taipei, 100225 Taiwan; 4 Department of Orthopedics, Taipei Tzu Chi Hospital, No. 289, Jianguo Rd., Xindian Dist, New Taipei, 231405 Taiwan

**Keywords:** Dorsal approach, Hammer toe deformity, Lesser metatarsophalangeal joint instability, Metatarsalgia, Plantar plate tear

## Abstract

**Background:**

The plantar plate is an important static stabilizer of the lesser metatarsophalangeal joints, and disruptions of the plantar plate can lead to significant instability and lesser toe deformities. In recent years, direct plantar plate repair has been proposed. Although direct repair via a dorsal approach is attractive, a torn plantar plate is small and difficult to access using regular instruments in a restricted operative field.

**Methods:**

In this report, a unique method for plantar plate repairs was used to repair various configurations of plantar plate tears with standard operative instruments that are available in most operating rooms.

**Results:**

Using this method, 10 patients underwent plantar plate repairs, and the mean follow-up period was 24 (range, 14–38) months. The mean visual analog scale score for pain preoperatively was 4.1 (range, 0–6) and decreased to 0.6 (range, 0–3) at last follow-up. Postoperatively, the mean visual analog scale score for satisfaction was 9.6 (range, 8–10) and the mean American Orthopedic Foot and Ankle Society forefoot score was 88.8 (range, 75–100).

**Conclusions:**

Our study proposes an inexpensive and versatile method for plantar plate repair via a dorsal approach that uses standard operative instruments.

**Trial registration:**

ClinicalTrials.gov, NCT04949685. July 2, 2021 - Retrospectively registered,

**Level of clinical evidence:**

4

**Supplementary Information:**

The online version contains supplementary material available at 10.1186/s12891-021-04951-w.

## Background

The plantar plate of a lesser metatarsophalangeal joint (MTPJ) is a thickening of the plantar part of the joint capsule with an average length of 19 mm and a thickness of 2 mm [1, 2]. The plantar plate is an important static stabilizer of MTPJ such that a disruption in it leads to significant instability and lesser toe deformities [3–7]. When chronic metatarsalgia is unrelieved or the lesser toes progressively deform, one potential course of action is operative treatment [3], which can include indirect reconstruction or direct repair. Methods for indirect reconstruction include extensor tendon lengthening, flexor tendon transfer, peri-articular soft tissue release, collateral ligament reconstruction, tenodesis, and metatarsal shortening osteotomy [4, 8–18].

In recent years, various methods of direct plantar plate repair have been proposed, whereby the attenuated distal part of the plantar plate is re-attached to its insertion on the base of the proximal phalanx of the lesser toe [9, 19–23]. Repair of the plantar plate can be achieved through a plantar incision. However, if the plantar plate tear is difficult to find through this approach, another dorsal incision can be made to identify the defect [19]. As there is a risk of a painful plantar scar forming in this case, a curvilinear incision with careful dissection is suggested [24, 25]. Direct repair via the dorsal approach is another option and several studies have reported satisfactory results [21, 22, 24, 26].

A torn plantar plate is small and difficult to access using the regular instruments in a restricted operative field via the dorsal approach [27, 28]. There are some commercial devices or instruments available for direct plantar plate repair, but they are costly and cannot be universally applied [24, 29]. Therefore, in this report, an inexpensive and versatile method for plantar plate repair was used to remedy various configurations of plantar plate tears using the standard equipment available in most operating rooms. Our small series was not intended to show the comprehensive results of plantar plate repair for lesser toe deformities. Rather, the feasibility and safety of this particular technique was explored.

## Methods

After receiving approval from the Institutional Review Board, a retrospective study was performed on patients who underwent operations for lesser MTPJ instability between September 2015 and December 2019. The operative indications for plantar plate repair were symptomatic MTPJ instability and clinical Grades of II to III, which were determined using the drawer test [20, 23]. Participants were aged between 20 and 90 years old at the time of their operation and had received plantar plate repair by the senior author (PYC). Exclusion criteria were previous operative treatment of the affected lesser MTPJ, patients with rheumatoid arthritis and neurologic disorders, and postoperative follow-ups of less than 12 months.

Radiographs were obtained, and the hallux valgus angle, 1–2 intermetatarsal angle, and MTPJ angle (positive angle indicates a lateral inclination, and negative angle denotes a varus inclination) were measured. The outcome analysis included the Lesser Metatarsophalangeal-Interphalangeal Scale from the American Orthopedic Foot and Ankle Society (AOFAS) forefoot score, the Visual Analogue Scale score for pain and satisfaction, and answering the question “Would you recommend this procedure to other patients?” [30, 31].

### Operative method

**Fig. 1:**
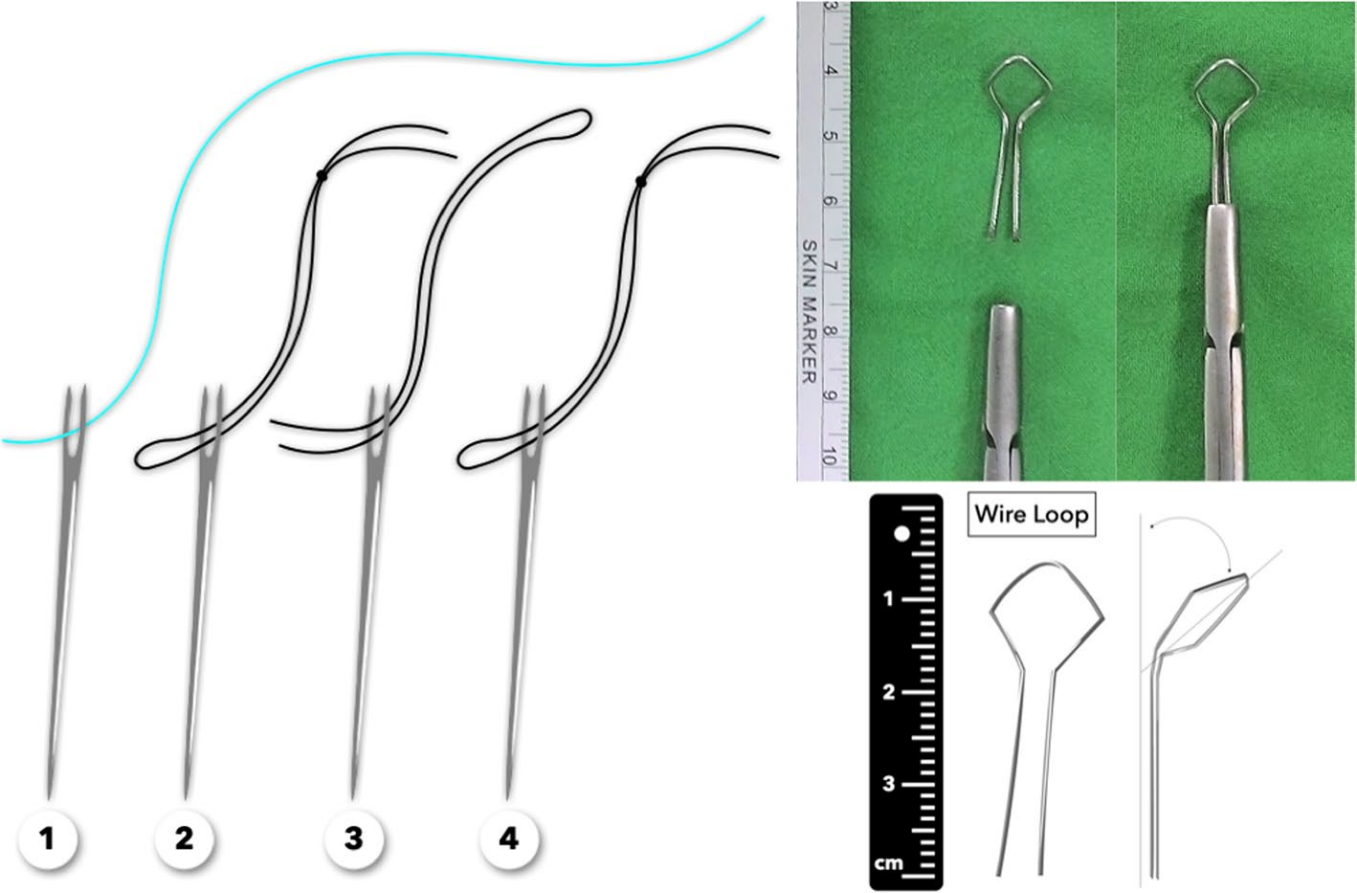
The equipment needed for this method included four straight needles, three 3–0 silk sutures, one 2–0 Ticron suture, and one 1.0-mm Kirschner-wire

**Fig. 2:**
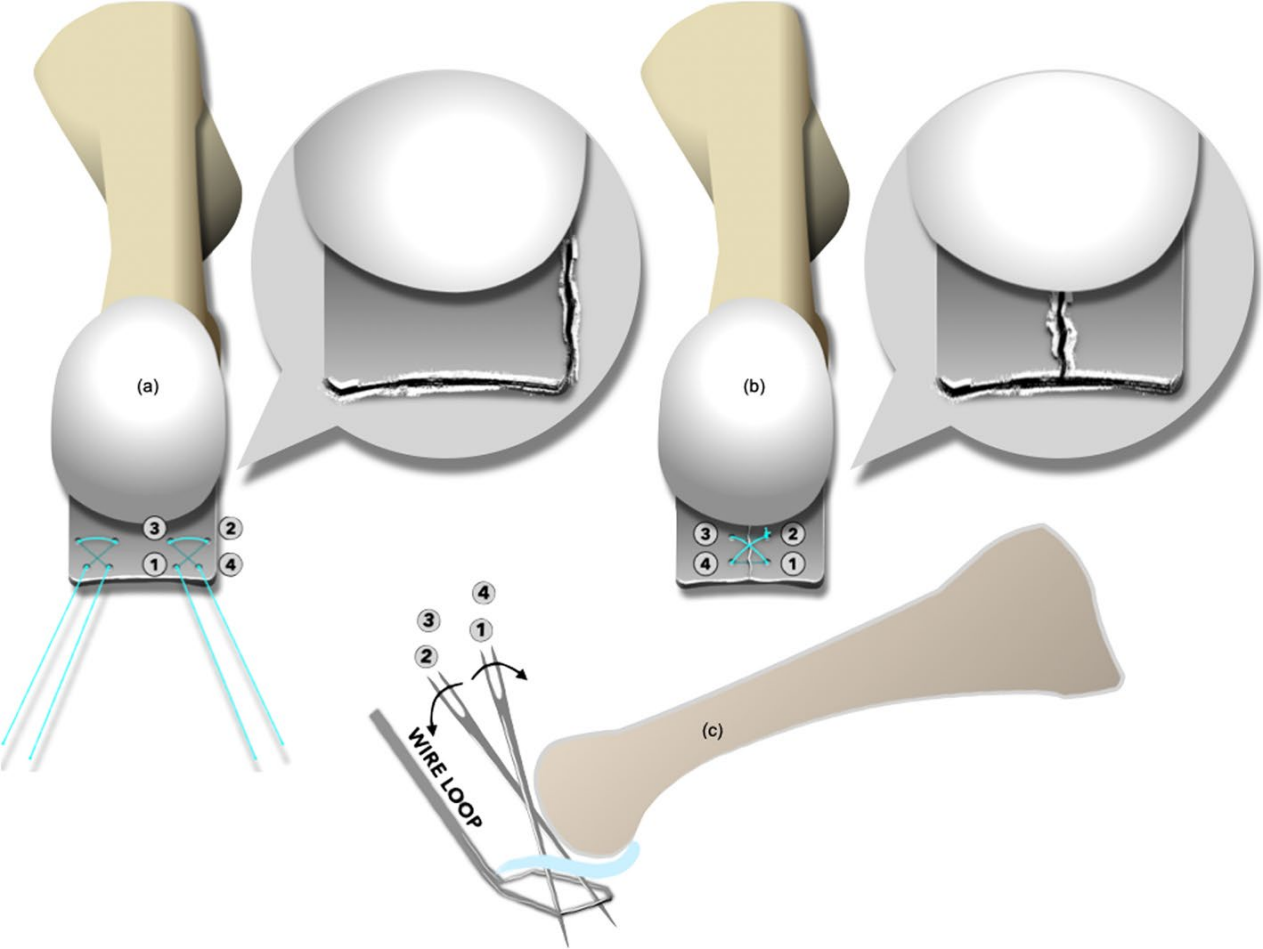
**a** Two sets of sutures were used in anatomical Grade II transverse tears or Grade III, “7”-type tears. **b** An anatomical Grade III, “T”-type tear could be converted into a transverse tear. **c** Using a straight needle makes it easier to target any point in the operator’s sightline, even in a considerably restricted field

The equipment needed for this method included four straight needles, three 3–0 silk sutures, one 2–0 Ti-Cron™(Metronic, Minneapolis, MN) braided polyester nonabsorbable suture (a 1–0 absorbable, synthetic, braided suture could also be used instead), and one 1.0-mm Kirschner wire. The wire loop, made of 1.0-mm Kirschner wire, was divided into two parts: the wire loop itself and the handle. The wire loop could be a circular or diamond shape with a diameter of around 8 mm, while the handle should be long enough to be steadily held by a wire twister. The wire loop and the handle were angulated around 45 degrees to facilitate insertion between the plantar plate and the flexor tendons (Fig. 1). A typical set of suture consists of four passes conducted by four straight needles. Using a straight needle makes it easier to target any point in the operator’s sightline, even in a considerably restricted field (Fig. 2).

**Fig. 3:**
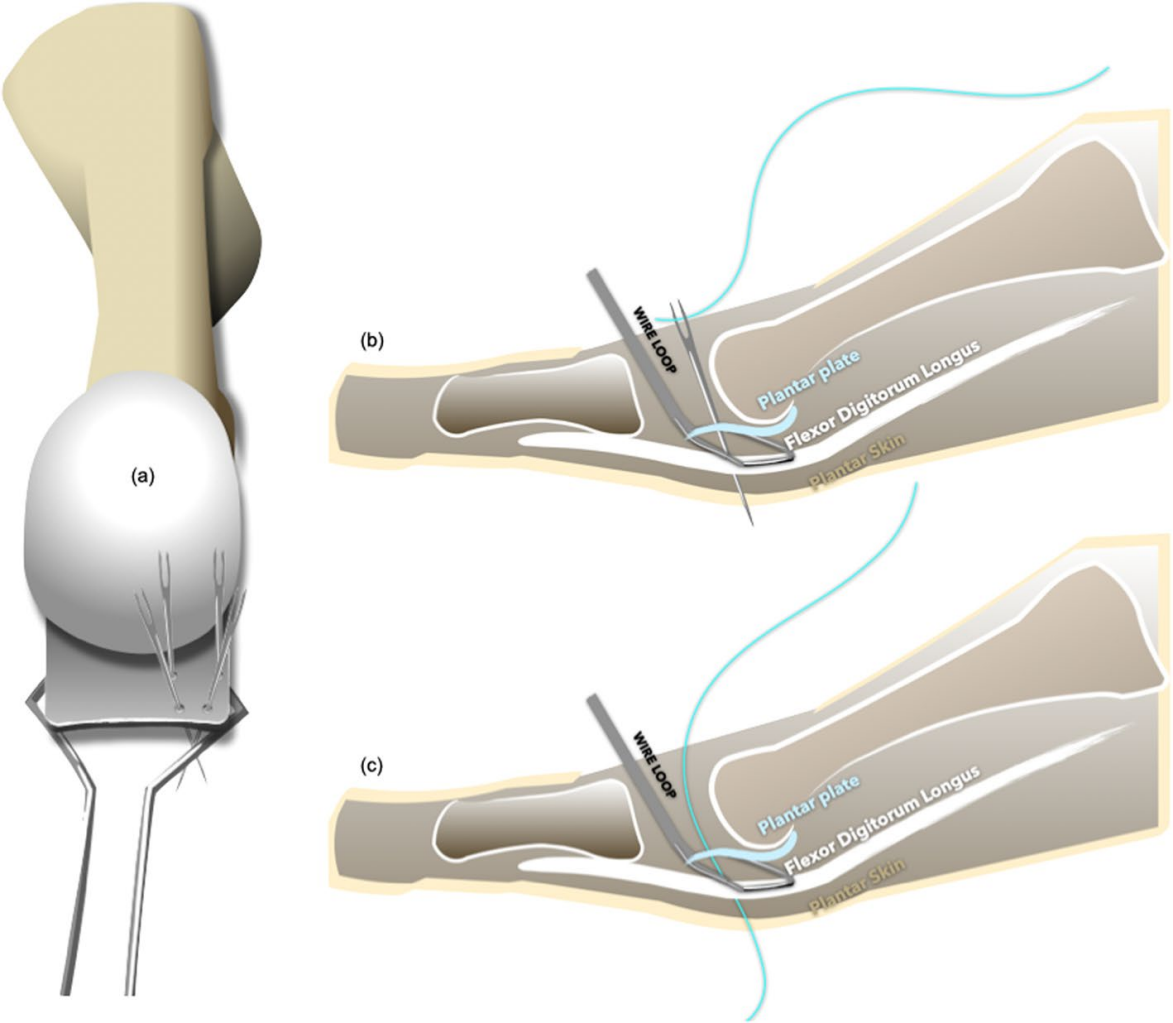
**a** our straight needles were sequentially put through the plantar plate, and all of them should be located within the wire loop. **b** Each needle was driven through the plantar skin after threading. **c** The suture was detached from the needle after passing through the plantar skin

After undergoing general anesthesia, patients were placed in a supine position with a bump beneath the ipsilateral hip. The leg was exsanguinated with a rubber bandage and the pneumatic tourniquet was inflated. A longitudinal skin incision was made over the dorsal aspect of the lesser MTPJ and the extensor tendons were retracted laterally to expose the joint capsule. After an incision was made in the dorsal capsule, the joint was distracted manually to inspect the plantar plate. When repair of the plantar plate was deemed necessary, the soft tissue attachments were released sequentially from the phalangeal base to expose the proximal phalangeal base. The length of the second metatarsal was measured using the Hardy and Clapham method preoperatively [32]. If a long metatarsal was present and shortening of the metatarsal was desirable, a Weil osteotomy could be performed to expose the plantar plate more easily. The suture retrieving loop was placed between the plantar plate and the flexor digitorum longus tendon after sequential dissections. Four straight needles were sequentially put through the plantar plate at the desired spots within the wire loop (Fig. 3). By aiming the needles slightly convergent toward the long axis of the corresponding ray, the risk of neurovascular injury could be minimized.

**Fig. 4:**
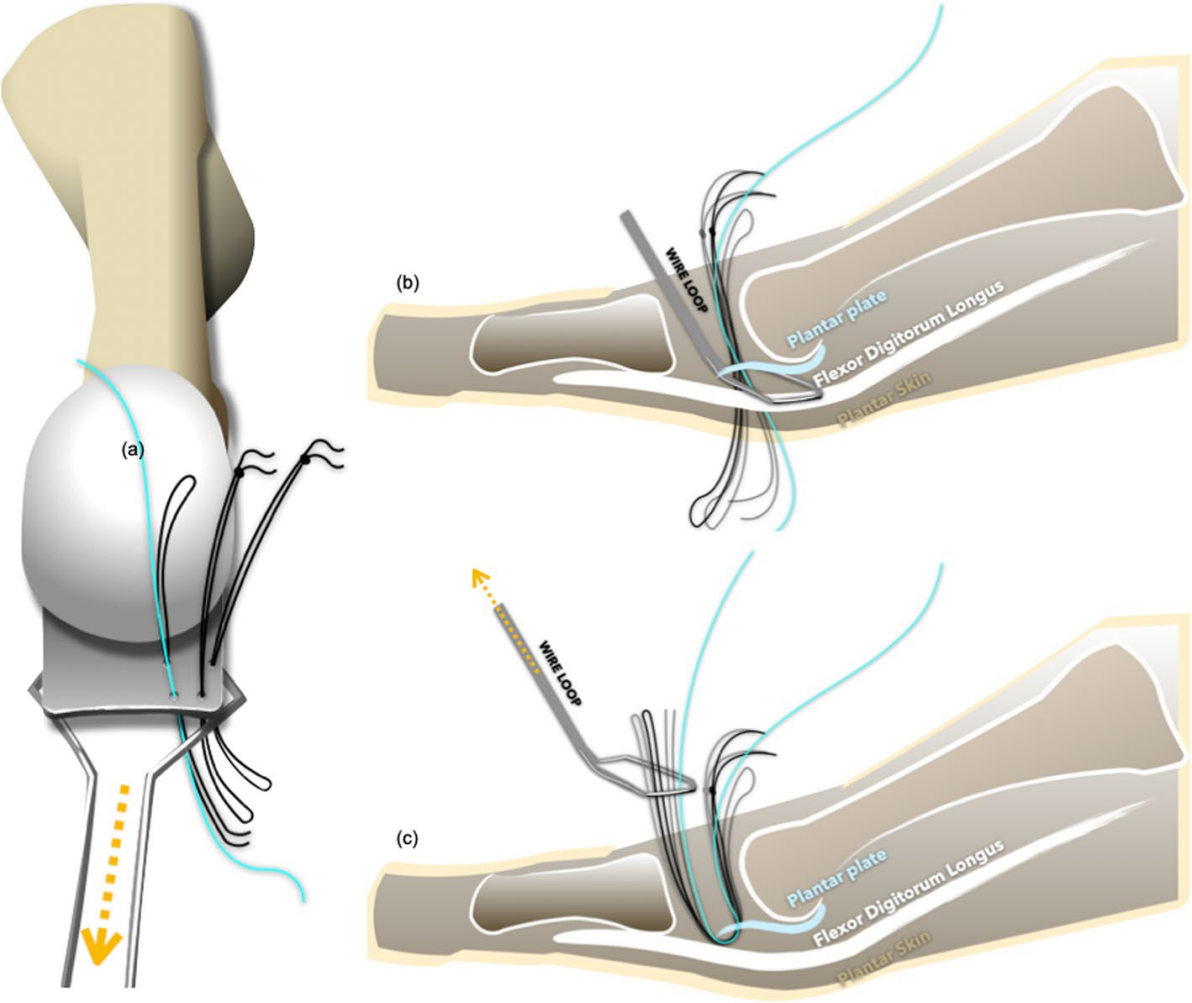
**a**-**b** Sutures and suture loops were passed to the plantar side. **c** As the suture retrieving loop was withdrawn, the distal end of the suture and suture loops were retrieved from the plantar skin; both ends came out from the dorsal aspect

The straight needles were threaded with one 2–0 Ti-Cron™ suture and three silk suture loops. Each needle was driven through the plantar skin. The suture was then detached from the needle after it passed through the plantar skin. After passing four needles through the plantar skin, the proximal ends of the suture and suture loops were kept at the dorsal aspect and the distal ends came out from the plantar aspect. As the wire loop was withdrawn, the distal ends of the suture and suture loops were retrieved, coming out of the wound dorsally (Fig. 4).

**Fig. 5:**
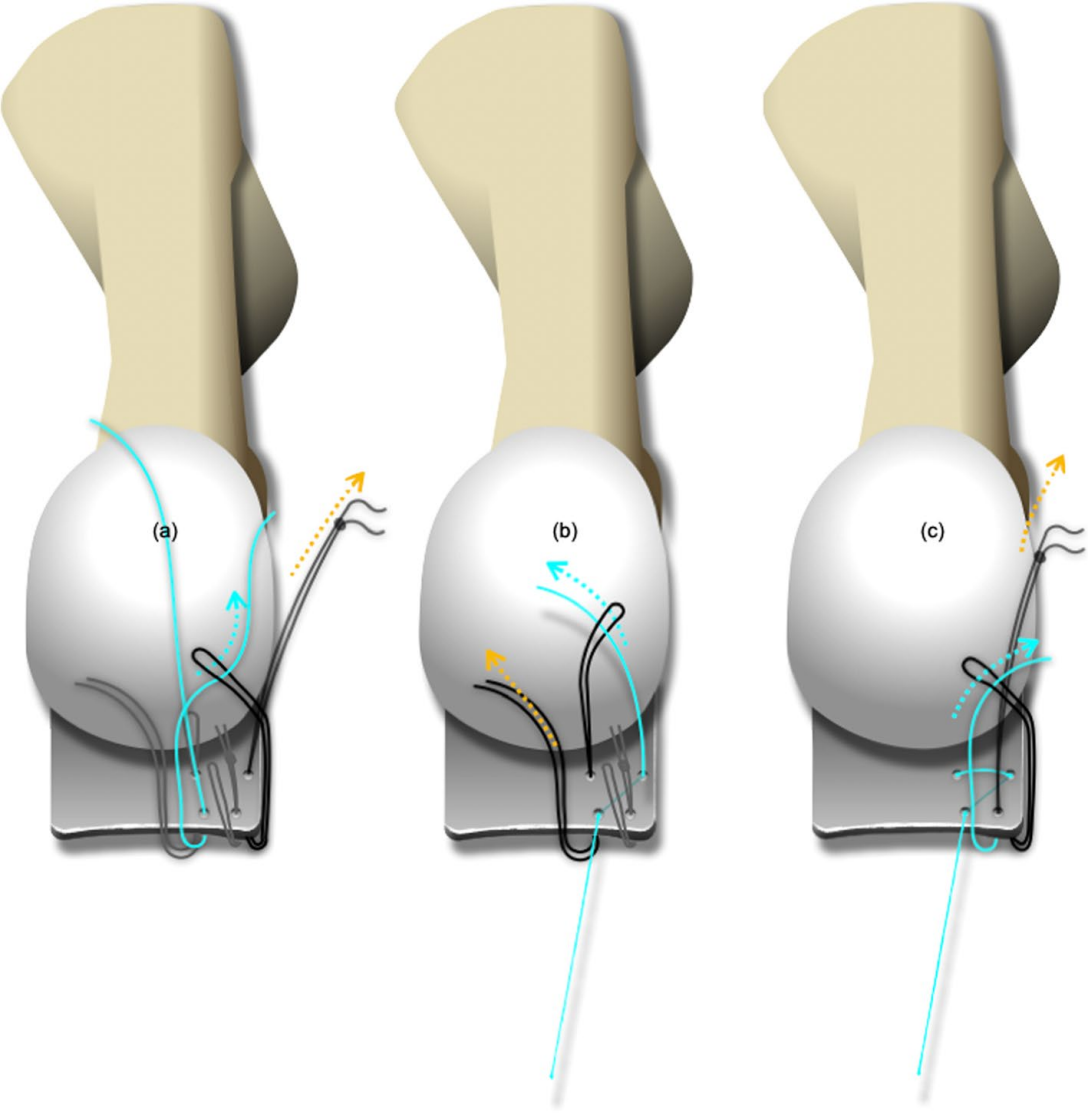
**a**-**c** The free end of the suture was passed through the plantar plate (from the dorsal to the plantar or from the plantar to the dorsal) with the aid of suture loops

**Fig. 6:**
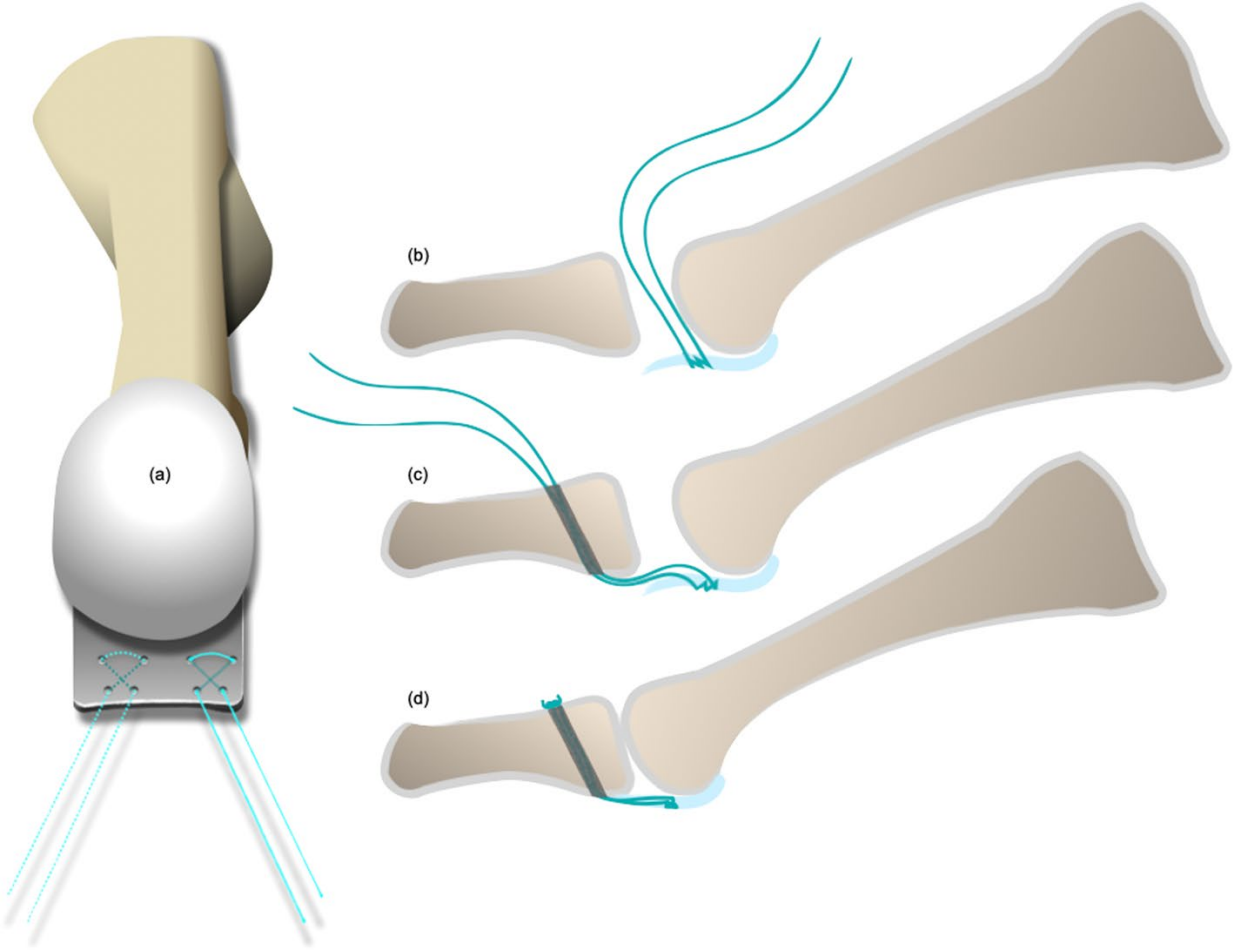
**a** Typically, two sets of sutures were used in anatomical Grade II transverse tears or Grade III, “7”-type tears. **b**-**d** Both ends of the sutures were then passed from the plantar to the dorsal through two tunnels, and the metatarsophalangeal joint could be reduced by tightening the sutures

**Fig. 7:**
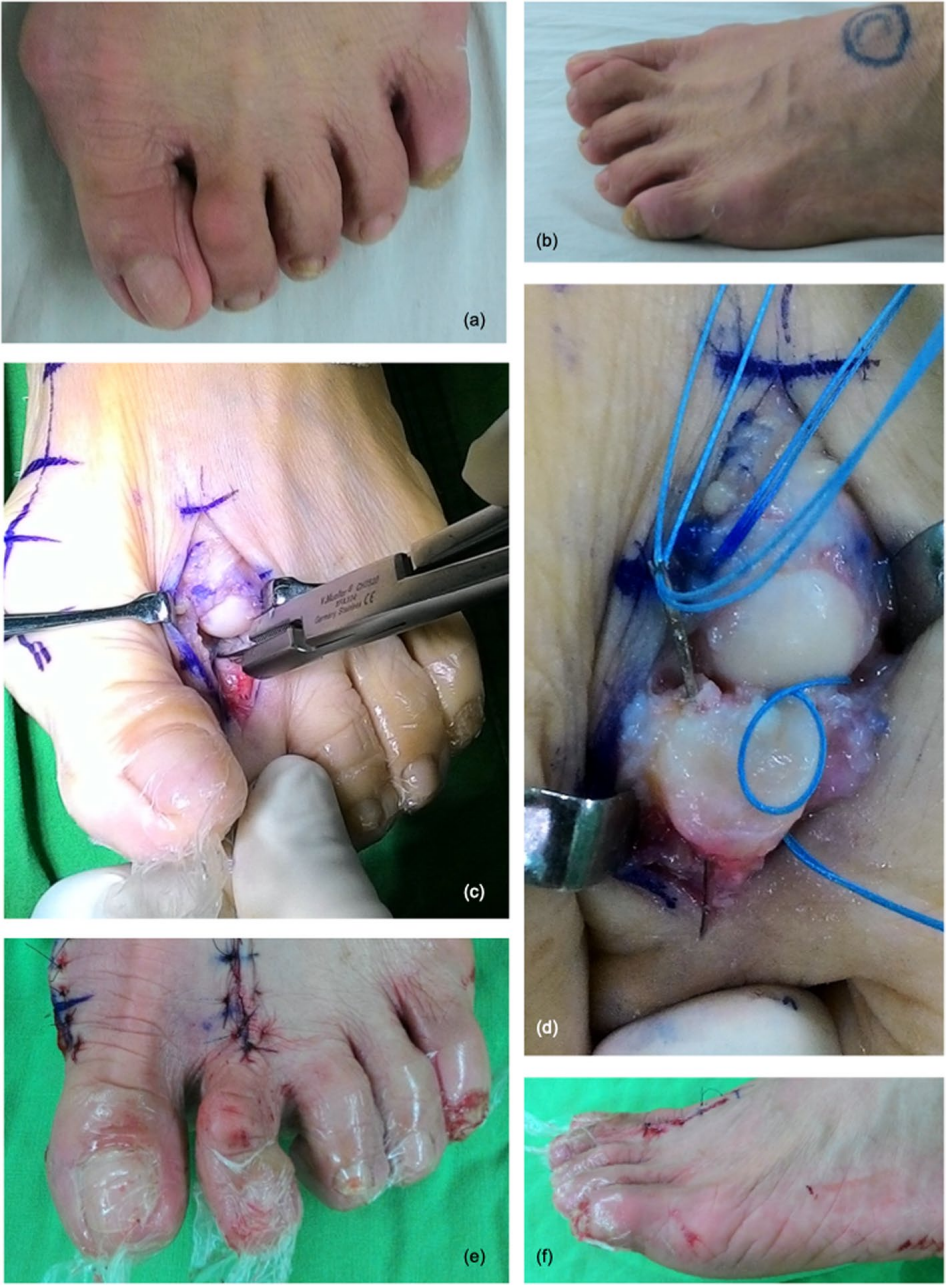
**a**-**b** The operative indications for plantar plate repair were symptomatic lesser metatarsophalangeal joint instability and most of the patients received concomitant operations for hallux valgus. **c** The suture retrieving loop was placed between the plantar plate and the flexor digitorum longus tendon. **d** Several clefts in the cortical bone of the distal insertion of the plantar plate were made to facilitate re-attachment of the plantar plate, and two tunnels were created to receive the sutures. **e**-**f** The hammer toe deformity was corrected after operation

The free end of the Ti-Cron™ suture was passed through the plantar plate (from the dorsal to the plantar or from the plantar to the dorsal) with the aid of the suture loops (Fig. 5). After completing the technique, there should have been a set of figure-of-eight suture within the plantar plate. The straight needles could be inserted at the desired spots in the plantar plate, and the procedure could be repeated several times depending on the configuration of the lesion and the adequacy of the sutures’ holding power. Typically, two sets of sutures were used in an anatomical Grade II transverse tear or a Grade III, “7″-type tear to reattach the plantar plate to the proximal phalanx [23]. An anatomical Grade III, “T”-type tear required an additional set of sutures to convert the “T”-type tear into a transverse tear (Fig. 2) [23]. Several clefts in the cortical bone of the distal insertion of the plantar plate (the plantar aspect of the proximal phalangeal base) were made using a rongeur or a bone cutter to facilitate re-attachment of the plantar plate, and two tunnels were drilled using a 1.2-mm Kirschner wire. Both ends of the sutures were then passed from the plantar to the dorsal through these two tunnels, and the sutures were tightened with the MTPJ in reduced position (Fig. 6) (Fig. 7). The repair can be protected either by a transarticular pin or by postoperative taping.

The postoperative protocol was as follows: removal of stitches after 2 weeks, removal of the trans-articular pin after 4 weeks, and protected weight bearing for 6 weeks. The operated toe was fixed in a plantarflexed position with surgical tapes for 8 weeks after the initial operation.

## Results

From September 2015 to December 2019, 18 patients underwent the plantar plate repair procedure. Of them, 10 patients (9 female and 1 male) met the inclusion and exclusion criteria. The mean age of these patients was 70 (range, 58–81) years old and the mean follow-up period was 24 (range, 14–38) months. Most of them received concomitant operations for hallux valgus or other foot pathologies (7 scarf osteotomies, 1 first MTPJ arthrodesis, 1 lateral column lengthening for flat foot, and 1 proximal metatarsal osteotomy), except one patient, who had an operation for lesser MTPJ instability only (Table 1). There were no instances of infection, ischemia, revision operations, or any other major complications related to the operations. Among these 10 patients, there were 11 lesser MTPJ (9 s toes and 2 third toes) that underwent plantar plate repair. All the lesser MTPJ operations were performed using the dorsal approach and the concomitant procedures performed along with the plantar plate repairs included 1 Weil osteotomy, 1 extensor tendon lengthening procedure, and 1 resection arthroplasty of the proximal interphalangeal joint.

**Table 1:** Patient results

Age	PPR	Concomitant procedure	Concomitant operation	Preoperative Pain (0–10)	Postoperative Pain (0–10)	AOFAS	SAT (0–10)
72	2			5	1		
58	2		Scarf	0	0	90	10
73	2,3		Scarf	6	0	90	10
67	3	ETL	Fusion, 2nd release	5	0	90	10
71	2	PIPR	Scarf	5	0	90	9
73	2		Scarf	3	1		
81	2		Scarf	4	1	75	10
73	2		Scarf, LCL	5	0	90	10
65	2		Scarf	3	0	100	10
66	2	Weil	PMO	5	3	85	8

*PPR* plantar plate repair, *ETL* extensor tendon lengthening, *PIPR* proximal interphalangeal joint resection arthroplasty, Fusion: arthrodesis for first metatarsal phalangeal joint, *LCL* lateral column lengthening for flat foot, *SAT* satisfaction, *PMO* proximal metatarsal osteotomy

The mean MTPJ angles preoperatively were 9.6 degrees laterally at the second MTPJ (range, − 7.7 to 34.8) and 8.9 degrees at the third MTPJ (range, − 6 to 25.9). Postoperatively, the mean MTPJ angles were 7.9 degrees laterally at the second MTPJ (range, − 5.1 to 26.8) and 9.1 degrees at the third MTPJ (range, − 2 to 20.2). At final follow up, the MTPJ angle shifted a mean of − 1.7 degrees at the second MTPJ (range, − 10.1 to 16.7) and 0.2 degrees at the third MTPJ (range, − 5.7 to 8.7) compared to the preoperative radiograph.

Improved clinical grading of the lesser MTPJ instability (Grade III to Grade I (*n* = 6), Grade II to Grade I (*n* = 2), and Grade II to Grade 0 (*n* = 3)) were achieved in all cases. Eight out of 10 patients agreed to participate in the postoperative outcome study and all of them stated they would recommend this procedure to other patients. The mean VAS score for pain preoperatively was 4.1 (range, 0–6) and decreased to 0.6 (range, 0–3) at last follow-up; the mean VAS score for satisfaction was 9.6 (range, 8–10); and the mean post-operative AOFAS forefoot score was 88.8 (range, 75–100) (Table 1).

### Discussion

The plantar plate is the major stabilizer of the MTPJ, and the relationship between the instability of lesser toes and plantar plate tears has been documented in the existing literature [3–7]. Such tears usually manifest as a chronic degenerative condition, but it can also occur after an acute injury [23, 33–36]. Progressive dorsal subluxation of an MTPJ, hammer toe deformity, cross over toes or elevated forefoot plantar pressure may occur after chronic attenuation of the plantar plate [3, 5, 7, 23, 37–43]. A clinical staging system has been proposed to quantify the MTPJ instability on a scale of 0 to 4. Grade 0 is a stable joint, Grade I is mild instability (< 50% subluxable), Grade II is moderate instability (> 50% subluxable), Grade III is gross instability (dislocatable joint), and Grade IV is a dislocated joint [20, 23]. Coughlin, Baumfeld, and Nery as well as Nery et al. proposed an anatomic grading classification based on the location, size, and shape of plantar plate tears, whereby a specific treatment method is chosen according to the grade awarded [20, 23]. Grade 0 is a plantar plate or capsular attenuation, Grade I is a transverse distal tear (< 50%), Grade II is a transverse distal tear (> 50%), Grade III is a transverse and/or longitudinal extensive tear, and Grade IV is an extensive tear with a buttonhole (dislocation).

Operative treatment of instability to the MTPJ has been proposed for almost 3 decades [4]. Direct repair of the plantar plate tear has received increased attention in recent years, and various methods have been proposed [9, 15, 19–23, 26, 44, 45]. The technique of direct plantar plate repair was initially suggested by Jolly and Ford et al. [9, 46] The dorsal approach prevents potential painful plantar scars and wound healing complications, and also provides the ability to perform metatarsal osteotomies and other soft tissue procedures. Approximately 5 mm of the plantar plate can be visualized after a sequential dorsal dissection of the second MTPJ, and 8 mm of the plantar plate can be visualized with the addition of a Weil osteotomy [27, 28]. Nery et al. prospectively enrolled 68 patients (100 MTPJs) with a mean follow-up period of 24 (range, 12–48) months treated according to the anatomical grade system for plantar plate tears [26]. Of these 100 MTPJs, 48 MTPJs underwent a Weil osteotomy followed by a direct repair of the plantar plate for Grades II and III, and the mean post-operative AOFAS score was 86.8 [26, 44]. However, visualization of the plantar plate and passing sutures for the plantar plate repair are still the most difficult part of the dorsal approach.

Some studies have proposed using suture passing techniques for plantar plate repairs [29, 47–49]. Clement et al. reported a technical tip of direct plantar plate repairs using a single skin hook to distract the distal end of the plantar plate and passing sutures with small-radius curved needles [29]. Clements and Ghai reported the case of a plantar plate injury that was treated using Keith needles to pass sutures through the plantar plate and a bent angiocatheter needle as a hook to retrieve the suture [48]. Kindred et al. reported a case series of six patients (9 joints), with a median follow-up of 19 (range, 19–39) months, that underwent plantar plate repairs using a similar method, whereby an angiocatheter was used as a passage-way to pass the suture [49]. Both reports offer methods to pass and retrieve suture through the plantar plate to repair a simple transverse tear without commercially available suture passing systems.

In this report, a unique method was proposed to retrieve sutures from a restricted operative field without using a commercial suture passing system. In contrast to the methods mentioned above, the suture could be passed through the plantar plate multiple times until enough holding power was achieved. By carefully targeting on the plantar plate using straight needles, an optimal configuration of the sutures could be applied. Anatomical Grade II or Grade III, “7”-type tears could be repaired with two sets of sutures. Although a Grade III, “T”-type tear might be difficult to repair conventionally or using a commercial suture passing system, it could readily be repaired using our method. A set of figure-of-eight suture was first applied on both sides of the “T”-type tear. A Grade III tear would then be turned into a Grade II simple transverse tear and it could be repaired in the same manner (Fig. 2b). Moreover, this method eliminated the risk of inadvertent suture to the underlying flexor tendons and neurovascular bundles. Our results showed no neurovascular complications related to this procedure in all the 18 patients. The present study was limited by the potential confounding factors inherent to retrospective studies, and the study findings were weakened by the small case numbers, the lack of preoperative comparison data, the lack of objective parameters for the operative outcome, and the lack of a control group. As most patients received concomitant operations for bunion, it is difficult to tell if the satisfaction was due to the hallux valgus corrections or the plantar plate repairs. However, since the efficacy of similar plantar plate repair via dorsal approach has been discussed in the literature, our present report focuses on demonstrating the versatility and safety of the novel suture passing technique.

### Conclusions

Our study demonstrated a versatile method for plantar plate repairs using the dorsal approach with standard operative instruments. Appropriate satisfaction rate and clinical outcomes were achieved during the short-term follow-up period without procedure-related complications.

## Supplementary Information

**Additional file 1.** Operative technique video.

**Additional file 2.** Raw data, supplementary information.

## Data Availability

The datasets used and/or analysed during the current study are available from the corresponding author on reasonable request.

## Abbreviations

MTPj: Metatarsophalangeal joint
AOFAS: American Orthopedic Foot and Ankle Society
PPR: Plantar plate repair
ETL: Extensor tendon lengthening
PIPR: Proximal interphalangeal joint resection arthroplasty
LCL: Lateral column lengthening for flat foot
SAT: Satisfaction
PMO: Proximal metatarsal osteotomy
